# Assessment of Safety and Outcome of Lateral Hypothalamic Deep Brain Stimulation for Obesity in a Small Series of Patients With Prader-Willi Syndrome

**DOI:** 10.1001/jamanetworkopen.2018.5275

**Published:** 2018-11-30

**Authors:** Ruth R. Franco, Erich T. Fonoff, Pedro G. Alvarenga, Eduardo J. L. Alho, Antonio Carlos Lopes, Marcelo Q. Hoexter, Marcelo C. Batistuzzo, Raquel R. Paiva, Anita Taub, Roseli G. Shavitt, Euripides C. Miguel, Manoel J. Teixeira, Durval Damiani, Clement Hamani

**Affiliations:** 1 Children’s Institute, Division of Pediatric Endocrinology, University of São Paulo Medical School, São Paulo, Brazil; 2 Division of Functional Neurosurgery of Institute of Psychiatry, Department of Neurology, University of São Paulo Medical School, São Paulo, Brazil; 3 Institute of Psychiatry, Department of Psychiatry, University of São Paulo Medical School, São Paulo, Brazil; 4 Harquail Centre for Neuromodulation, Division of Neurosurgery, Sunnybrook Health Sciences Centre, University of Toronto, Toronto, Ontario, Canada

## Abstract

**Importance:**

Deep brain stimulation (DBS) has been investigated for treatment of morbid obesity with variable results. Patients with Prader-Willi syndrome (PWS) present with obesity that is often difficult to treat.

**Objective:**

To test the safety and study the outcome of DBS in patients with PWS.

**Design, Setting, and Participants:**

This case series was conducted in the Hospital das Clínicas, University of São Paulo, Brazil. Four patients with genetically confirmed PWS presenting with severe obesity were included.

**Exposure:**

Deep brain stimulation electrodes were bilaterally implanted in the lateral hypothalamic area. After DBS implantation, the treatment included the following phases: titration (1-2 months), stimulation off (2 months), low-frequency DBS (40 Hz; 1 month), washout (15 days), high-frequency DBS (130 Hz; 1 month), and long-term follow-up (6 months).

**Main Outcomes and Measures:**

Primary outcome measures were adverse events recorded during stimulation and long-term DBS treatment. Secondary outcomes consisted of changes in anthropometric measures (weight, body mass index [calculated as weight in kilograms divided by height in meters squared], and abdominal and neck circumference), bioimpedanciometry, and calorimetry after 6 months of treatment compared with baseline. The following evaluations and measurements were conducted before and after DBS: clinical, neurological, psychiatric, neuropsychological, anthropometry, calorimetry, blood workup, hormonal levels, and sleep studies. Adverse effects were monitored during all follow-up visits.

**Results:**

Four patients with PWS were included (2 male and 2 female; ages 18-28 years). Baseline mean (SD) body mass index was 39.6 (11.1). Two patients had previous bariatric surgery, and all presented with psychiatric comorbidity, which was well controlled with the use of medications. At 6 months after long-term DBS, patients had a mean 9.6% increase in weight, 5.8% increase in body mass index, 8.4% increase in abdominal circumference, 4.2% increase in neck circumference, 5.3% increase in the percentage of body fat, and 0% change in calorimetry compared with baseline. Also unchanged were hormonal levels and results of blood workup, sleep studies, and neuropsychological evaluations. Two patients developed stimulation-induced manic symptoms. Discontinuation of DBS controlled this symptom in 1 patient. The other required adjustments in medication dosage. Two infections were documented, 1 associated with skin picking.

**Conclusions and Relevance:**

Safety of lateral hypothalamic area stimulation was in the range of that demonstrated in patients with similar psychiatric conditions receiving DBS. In the small cohort of patients with PWS treated in our study, DBS was largely ineffective.

## Key Points

**Question:**

Is lateral hypothalamic deep brain stimulation (DBS) safe for the treatment of obesity in Prader-Willi syndrome?

**Findings:**

In this case series of 4 patients with Prader-Willi syndrome treated with lateral hypothalamic DBS, 2 developed stimulation-induced manic symptoms and 2 developed infections, 1 of which was associated with skin picking. No major differences were found when postoperative anthropometric and calorimetric data were compared with baseline; hormonal levels, and results of blood workup, sleep studies, and neuropsychological evaluations also showed no change.

**Meaning:**

Safety of lateral hypothalamic DBS was in the range of that demonstrated in patients with similar psychiatric conditions receiving DBS; however, in the small cohort of patients with Prader-Willi syndrome treated, DBS seemed largely ineffective for the treatment of obesity.

## Teaser

This study of a case series of 4 patients investigates the safety and efficacy of deep brain stimulation for obesity associated with Prader-Willi syndrome.

## Introduction

Obesity is one of the hallmarks of Prader-Willi Syndrome (PWS) and is largely refractory to medical and surgical therapies in this context.^1^ Preclinical evidence suggests that hypothalamic stimulation may reduce food consumption and weight gain.^2,3,4,5^ In the clinic, a patient with morbid obesity treated with hypothalamic DBS had transiently lost 12 kg over 5 months.^6^ Two of 3 patients receiving lateral hypothalamic area (LHA) DBS had significant increases in metabolism at rest.^7^

We report the safety and outcome data on the use of LHA DBS in 4 patients with PWS.

## Methods

### Patients

This nonrandomized study was approved by the research ethics board of the Hospital das Clínicas, University of São Paulo, Brazil, and registered at ClinicalTrials.gov (NCT02297022). Written informed consent was obtained from legally authorized representatives. The study largely followed the Transparent Reporting of Evaluations With Nonrandomized Designs (TREND) reporting guideline for nonrandomized trials, except for a few items not applicable to our study (eg, statistical analyses, allocation, patients lost to follow-up, comparison with target population, intention-to-treat analysis).

Inclusion criteria were (1) age 15 years or older; (2) diagnosis confirmed by genetic analysis; (3) severe obesity, defined as body mass index (BMI [calculated as weight in kilograms divided by height in meters squared]) greater than 35 or BMI greater than 30 in patients kept on a strict diet; and (4) legal guardians able to sign the consent. Exclusion criteria were (1) cardiovascular symptoms that could increase surgical risk; (2) clinically relevant brain abnormalities; (3) implanted stimulators; (4) medical condition requiring body magnetic resonance imaging; (5) history of seizures; (6) plans to use diathermy; and (7) currently participating in another trial.

In total, 5 patients treated in the Division of Pediatric Endocrinology of the Hospital das Clínicas, University of São Paulo, Brazil, were screened by investigators participating in the trial. All received growth hormone during childhood. Patients were living at home with their parents and participated regularly in daily activities. One patient was excluded because his BMI was below 30. Procedures and follow-up appointments were conducted in the Hospital das Clínicas, University of São Paulo, Brazil.

### Baseline Evaluations

Baseline evaluations included clinical and neurological examinations, psychiatric and neuropsychological assessments, anthropometry (weight, BMI, and abdominal and neck circumference), bioimpedanciometry (Quantum II; RJL Systems, Inc), and calorimetry (CPX/Ultima; MedGraphics). Blood samples to measure the following hormones were collected: growth hormone, follicle stimulating hormone, luteinizing hormone, testosterone, estradiol, cortisol, adrenocorticotropic hormone, prolactin, thyroid-stimulating hormone, free thyroxine, insulin, and insulin-like growth factor 1. Blood workup included measurements of cholesterol and fractions (high-density lipoproteins and low-density lipoproteins), triglycerides, glucose, aspartate aminotransferase, alanine aminotransferase, γ-glutamyltransferase, 25-hydroxyvitamin D, and uric acid. Additional examinations were electrocardiogram, echocardiogram, chest radiography, abdominal ultrasound, magnetic resonance imaging, and sleep studies.

Primary outcome measures were adverse events recorded during stimulation and chronic DBS treatment. Secondary outcomes consisted of changes in anthropometric measures, bioimpedanciometry, and calorimetry after 6 months of chronic DBS compared with baseline. Neither patients nor investigators were blinded as to whether stimulation was being delivered.

### Surgical Procedure

Surgery was conducted 2 to 4 weeks after baseline assessments (eMethods in the Supplement). The selected target was adjacent to the fornix, anterolateral to the mammillary bodies, and posterior to the optic tract.^8^ No microrecordings were used. Once electrodes were implanted (model 6149; St Jude Medical), contacts were tested for adverse effects. Stimulation of ventral contacts at 130 Hz, 91 μsec, and 3.5 mA or greater induced significant increases in heart rate with no concomitant changes in blood pressure. While receiving stimulation, patients did not notice changes in hunger or satiety. No memory flashbacks or experiential phenomena were recorded. After testing, electrodes were connected to extension cables externalized through the scalp. A computed tomography scan was then obtained. Five days later, electrodes were connected to a pulse generator (Libra XP6644; St Jude Medical) under general anesthesia.

To study the location of contacts used for chronic stimulation, postoperative computed tomography merged to preoperative T1 images were morphed to the USP-Würzburg brain atlas^9^ (eMethods in the Supplement).

### Postoperative Care

Stimulation was commenced 10 days after surgery at 1.5 mA, 91 μsec, and 40 Hz, a frequency in the range of that associated with weight loss in our previous report.^6^ Selected cathodes were ventral contacts (case as anode). Patients were initially assessed every 3 to 7 days. If no changes in hunger or weight were noticed, stimulation was increased by 0.5 mA. Once 3 mA was reached, the frequency was increased to 130 Hz, followed by testing of the second most ventral contact. This titration phase lasted 1 to 2 months. Because patients 1 and 2 developed manic symptoms at 3.5 mA and patient 4 had a postoperative skin infection, DBS systems were only activated 2 months after titration (eFigure in the Supplement). Patients were initially given 1 month of low-frequency stimulation (3 mA, 210 μsec, and 40 Hz) followed by 15 days washout and 1 month of high-frequency stimulation (2 mA, 91 μsec, and 130 Hz). Thereafter, DBS was administered at either 40 or 130 Hz for 6 months (frequency selected based on subjective reports provided by caregivers). During chronic stimulation, patient 1 received 2 mA, 91 μsec, and 130 Hz (contacts 2 and 6 as cathodes and the case as anode). Patients 2, 3, and 4 received 3 mA, 210 μsec, and 40 Hz (contacts 1 and 5 as cathodes and the case as anode). Mean (SD) coordinates of the active contacts (midpoint of the cathodes) used for chronic stimulation were 8.3 (2.5) mm lateral, 5.8 (2.1) mm anterior, and 7.3 (1.7) mm inferior to the midcommissural point.

Anthropometric measures and clinical evaluations were obtained in the end of the off-stimulation, low-frequency stimulation, and high-frequency stimulation phases and 6 months after long-term stimulation. Calorimetry, bioimpedanciometry, neuropsychological assessments, hormonal levels, blood workup, and sleep studies were conducted at baseline and during the 6-month follow-up visit. No activities were done to increase patient compliance.

### Statistical Analysis

Data in the text and tables are presented as mean (standard deviation). As only 4 patients were treated and our primary outcome was safety, no statistical analyses comparing preoperative and postoperative values were conducted. As this is the first study, to our knowledge, to deliver DBS to patients with PWS, power was not calculated because we could not predict the effect size.

## Results

Two male and 2 female patients with genetically documented PWS and age ranging from 18 to 28 years were included (Table 1). Mean (SD) baseline BMI was 39.6 (11.1). Two patients had previous bariatric surgery. All presented psychiatric comorbidities, including skin picking, nail biting, aggressive behavior, hypersexuality, episodes of hypomania, psychosis, and impulsiveness. These were well controlled with medications.

**Table 1.** Patient Demographic and Clinical Characteristics

Patient No.	Comorbidities		Preoperative Medications	Bariatric Surgery
	Clinical	Psychiatric		
1	Obesity; hypogonadotrophic hypogonadism; hepatic steatosis	Psychosis; hypomania	Clozapine; topiramate	Sleeve gastrectomy
2	Obesity; hyperphagia; hypogonadotrophic hypogonadism	Aggressiveness; hypersexuality; impulsiveness	Topiramate	Sleeve gastrectomy; Roux-en-Y gastric bypass
3	Obesity; hyperphagia; hypogonadotrophic hypogonadism	Aggressiveness	Periciazine; clonazepam	None
4	Obesity; hypogonadotrophic hypogonadism	Skin picking; nail biting	None	None

No major differences in preoperative and postoperative anthropometric and calorimetric data were recorded (Table 2). Six months after receiving DBS at best settings, patients had a mean 9.6% increase in weight, 5.8% increase in BMI, 8.4% increase in abdominal circumference, 4.2% increase in neck circumference, 5.3% increase in the percentage of body fat, and 0% change in calorimetry compared with baseline. Also unchanged were hormonal levels and results of blood workup, sleep studies, and neuropsychological evaluations (eTables 1-4 in the Supplement). Individualized data are shown in eTables 5 to 7 in the Supplement. Contacts used for chronic DBS had a volume of tissue activated that encompassed the LHA (Figure).

**Table 2.** Preoperative and Postoperative Anthropometry, Bioimpedanciometry, and Calorimetry

Outcome Measure	Mean (SD) [% Change From Baseline]				
	Baseline	DBS Off	LFS	HFS	6-mo Follow-up
Weight, kg	95.0 (27.6)	98.9 (26.1) [4.1]	97.6 (26.8) [2.8]	100.4 (25.3) [5.7]	104.1 (26.8) [9.6]
Body mass index^a^	39.6 (11.1)	38.5 (8.5) [−2.7]	39.3 (7.9) [−0.8]	40.4 (7.3) [2.1]	41.9 (7.8) [5.8]
Circumference, cm					
Abdominal	116.0 (19.9)	123.5 (14.9) [6.5]	120.8 (14.4) [4.1]	123.5 (14.5) [6.5]	125.8 (15.2) [8.4]
Neck	36.0 (6.3)	36.4 (5.8) [1.0]	37.6 (3.5) [4.5]	38.8 (3.8) [7.6]	37.5 (4.5) [4.2]
Bioimpedanciometry, %	49.9 (3.5)	NC	NC	NC	52.5 (3.1) [5.3]
Calorimetry, kcal	1764.0 (242.5)	NC	NC	NC	1764.8 (308.4) [0]

Abbreviations: DBS, deep brain stimulation; HFS, high-frequency stimulation; LFS, low-frequency stimulation; NC, not collected.

^a^ Calculated as weight in kilograms divided by height in meters squared.

**Figure.**
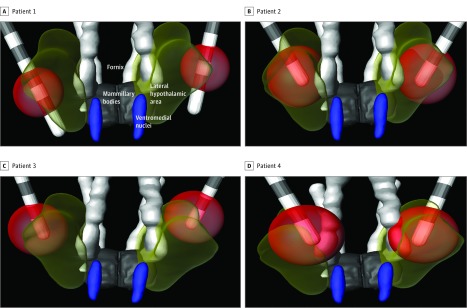
Schematic Representation of Electrode Location Postoperative computed tomography merged with preoperative T1 images were morphed to the USP-Würzburg atlas of the human brain and normalized to MNI standard space. The trajectory of the electrodes was determined and the volume of tissue activated calculated, as described in the text. Red spheres represent the volume of tissue activated when clinically used cathodes were stimulated at long-term deep brain stimulation settings (2 mA in patient 1 and 3 mA in patients 2, 3, and 4).

### Adverse Events

Patients 1 and 2 developed manic symptoms during the titration phase. Discontinuation of DBS was associated with symptomatic improvement in patient 1 but not in patient 2, who required an increase in topiramate. Patient 1 was receiving preoperative testosterone injections for hypogonadism, which infrequently resulted in priapism. A new episode occurred during the titration phase, requiring drainage.

Seven months after DBS, patient 1 developed an infection over the connector site likely due to skin picking. As DBS was largely ineffective, the family opted for removing the system. Patient 4 developed a superficial infection over the pulse generator during the first postoperative month that was successfully treated with antibiotics.

## Discussion

We found that safety of LHA stimulation was in the range of that demonstrated in patients with similar psychiatric conditions treated with DBS. In the small cohort of patients with PWS treated in our study, DBS seemed to be largely ineffective. Recorded adverse effects were hardware-related complications and manic symptoms, which might have occurred because of the recruitment of nearby structures (eg, medial subthalamic nucleus or the medial forebrain bundle).

Today, the classical concept of specific hypothalamic centers of appetite and satiety is outdated.^10^ This was supported by early experiments that contributed to our knowledge about mechanisms and circuits involved in these processes. Initial studies targeting the LHA in rodents^11,12^ and felines^13^ showed an increase in feeding, an effect partially attributed to the modulation of the medial forebrain bundle.^14^ In contrast, recent experiments in rodents reported a reduction in weight gain following high-frequency stimulation.^4,15^ Preclinical work in which the ventromedial nuclei were targeted has also been controversial. While some studies in rodents,^16,17,18,19^ dogs,^2^ minipigs,^3^ and nonhuman primates^5^ have shown altered feeding behavior, changes in type of food ingested, and/or a decrease in weight gain, others reported no substantial changes^20^ or even an increase in food consumption following stimulation.^21^

As feeding involves rewarding aspects, stimulation of the nucleus accumbens (NAc) has also been investigated in animal models. Obese animals receiving NAc shell stimulation had a reduction in weight gain^22^ associated with increased dopamine levels and the upregulation of D2 receptors.^10,22^ Reductions in binge eating have also been documented in rodents receiving NAc core^23^ and NAc shell stimulation,^24^ the latter effect being mitigated by D2 receptor antagonists.

To date, a few clinical studies have been conducted using DBS to treat patients with obesity. In 2008, a single patient was treated with hypothalamic DBS with electrodes implanted near the fornix.^6^ When stimulated at 50 Hz he lost 12 kg over 5 months.^6^ When required to turn off the device at night, the patient binged and regained the weight he had lost.^6^ In more recent work, 3 obese patients were treated with LHA stimulation and followed up for an average of 35 months.^7^ Deep brain stimulation significantly increased resting metabolic state in 2 patients but induced no substantial changes in weight. The outcome of NAc DBS was described in 2 recent trials. A patient who developed hypothalamic obesity following the onset of and surgery to treat a craniopharyngioma lost more than 13 kg in 14 months.^25^ Rezai and colleagues^26^ treated 3 obese patients with BMI greater than 40 for whom treatment with Rou-en-Y bypass failed. One patient successfully completed the DBS trial and lost 100 pounds. One patient required the system to be explanted, and the other committed suicide. The authors mentioned that all patients had a history of psychiatric comorbidity and were exposed to important psychosocial stressors during the trial. Deep brain stimulation itself was not thought to be the reason for study withdrawal or suicide.

Target selection in our study was based on preclinical^4,15^ and clinical^7^ evidence. In addition, the LHA has unique neural elements and anatomical connections, serving as an interface between food consumption and reward.^14^ In PWS, postmortem studies suggest a relative preservation of orexin-positive cells in LHA.^27^ As patients with PWS have increased plasmatic levels of orexin A,^28^ we hypothesized that the modulation of local neuronal firing following DBS could have reduced orexin levels and food intake.

### Limitations

Our study was preliminary in nature and not without limitations. Only 4 patients were included. Despite this fact, recorded changes after DBS were of small magnitude and little variability. The main exception was the documented reduction in testosterone. We believe this was not stimulation induced for several reasons. First, only 1 of 2 patients experienced this event. Second, similar hormonal variability had been documented in that same patient during pretrial examinations. This was attributed to the time of blood collection, which was always done in the morning but not in the same hour. Although the reduction of testosterone in patient 3 was approximately 60%, we note that both preoperative and postoperative levels were substantially lower than the normal value for men of the patient’s age, reflecting his hypogonadotropic hypogonadism. A second aspect that needs to be discussed is the selected hypothalamic target. It is possible that DBS delivered to different hypothalamic nuclei (eg, ventromedial)^2,3,5^ or brain structures (eg, NAc or ventral internal capsule) might have yielded different results.^25,26^ This is important if one takes into account neuroimaging studies showing an increased activation of reward circuits when patients with PWS are presented with food stimuli.^29^ A third aspect involves adverse effects. Stimulation-induced manic symptoms have been reported in patients with psychiatric disorders receiving DBS.^30,31^ In our study, this occurred only after a certain current threshold was reached. Manic symptoms were reversible in 1 patient following stimulation offset, while the other required medication adjustments. As for infections, patients with PWS may be at particular risk, especially when presenting comorbid skin picking.^32,33^ Although this diagnosis cannot be considered a surgical contraindication, its presence requires and extra degree of caution to avoid erosions and subsequent infections of the system. As a final remark, patients with PWS present hypothalamic dysfunction (although not particularly in the LHA) and structural abnormalities that might have precluded a positive therapeutic response.

In a recent study, we have recorded LHA activity in a patient with PWS and tested DBS effects during food intake.^8^ After eating, the patient described a sensation of fullness but continued to crave food. Rewarding components and craving are associated with a subjective desire to gain pleasure and not directly related to homeostatic feeding. We cannot rule out that hypothalamic stimulation might regulate physiological aspects without affecting craving, which would be an important mechanism for increased food intake in PWS.

## Conclusions

This study found that LHA DBS was relatively safe but largely ineffective for improving anthropometric measures in a cohort of 4 patients with PWS.

## Notes

Supplement. **eMethods.** Surgical Procedure and Location of Electrodes **eReferences** **eFigure.** Study Timeframe **eTable 1.** Pre- and Postoperative Hormonal Levels **eTable 2.** Pre- and Postoperative Levels of Lipids, Markers of Hepatic Function, Glucose, Insulin, Vitamin D and Uric Acid **eTable 3.** Pre- and Postoperative Sleep Studies **eTable 4.** Pre- and Postoperative Neuropsychological Assessments **eTable 5.** Individualized Pre- and Postoperative Results of Anthropometry, Bioimpedanciometry and Calorimetry **eTable 6.** Individualized Measures of Pre- and Postoperative Hormonal Levels **eTable 7.** Individualized Measures of Pre- and Postoperative Levels of Lipids, Markers of Hepatic Function, Glucose, Insulin, Vitamin D and Uric Acid
