# Supplementary material for: Assessment of Safety and Outcome of Lateral Hypothalamic Deep Brain Stimulation for Obesity in a Small Series of Patients With Prader-Willi Syndrome
Source: JAMA Netw Open. 2018 Nov 30;1(7):e185275. doi: 10.1001/jamanetworkopen.2018.5275 (PMC6324383; doi:10.1001/jamanetworkopen.2018.5275)
Supplement: Supplement. — eMethods. Surgical Procedure and Location of Electrodes eReferences eFigure. Study Timeframe eTable 1. Pre- and Postoperative Hormonal Levels eTable 2. Pre- and Postoperative Levels of Lipids, Markers of Hepatic Function, Glucose, Insulin, Vitamin D and Uric Acid eTable 3. Pre- and Postoperative Sleep Studies eTable 4. Pre- and Postoperative Neuropsychological Assessments eTable 5. Individualized Pre- and Postoperative Results of Anthropometry, Bioimpedanciometry and Calorimetry eTable 6. Individualized Measures of Pre- and Postoperative Hormonal Levels eTable 7. Individualized Measures of Pre- and Postoperative Levels of Lipids, Markers of Hepatic Function, Glucose, Insulin, Vitamin D and Uric Acid [file jamanetwopen-1-e185275-s001.pdf]

Supplementary Online Content

Franco RR, Fonoff ET, Alvarenga PG, et al. Assessment of safety and outcome of lateral hypothalamic deep brain stimulation for obesity in a small series of patients with Prader-Willi syndrome. *JAMA Netw Open*. 2018;1(7):e185275. doi:10.1001/jamanetworkopen.2018.5275

eMethods. Surgical Procedure and Location of Electrodes
eReferences
eFigure. Study Timeframe
eTable 1. Pre- and Postoperative Hormonal Levels
eTable 2. Pre- and Postoperative Levels of Lipids, Markers of Hepatic Function, Glucose, Insulin, Vitamin D and Uric Acid
eTable 3. Pre- and Postoperative Sleep Studies
eTable 4. Pre- and Postoperative Neuropsychological Assessments
eTable 5. Individualized Pre- and Postoperative Results of Anthropometry, Bioimpedanciometry and Calorimetry
eTable 6. Individualized Measures of Pre- and Postoperative Hormonal Levels
eTable 7. Individualized Measures of Pre- and Postoperative Levels of Lipids, Markers of Hepatic Function, Glucose, Insulin, Vitamin D and Uric Acid

This supplementary material has been provided by the authors to give readers additional information about their work.

eMethods

Surgical procedure

Surgery was conducted between 2-4 weeks after baseline assessments. First, a stereotactic frame was placed into the patient's head under local anesthesia and light sedation. Thereafter, patients underwent a stereotactic computed tomography scan. After the exam, images were exported to a neuronavigation station, merged with preoperative MRI series and used to determine the target region. The selected target was adjacent to the fornix, anterolateral to the mammillary bodies and posterior to the optic tract [1]. No microrecordings were used. Once implanted, the 4 contacts of each of the two leads (model 6149; St Jude Medical) were tested for side effects and efficacy (e.g. patients were asked how hungry their was on a scale from 0-10). DBS delivered to the deepest contacts (1 and 5 in electrodes numbered from 1-4 and 5-8) at 130Hz, 91µsec and ≥3.5mA induced significant increases in heart rate, with no concomitant changes in blood pressure. No changes in hunger or memory flashbacks were reported. After testing, electrodes were connected to externalized extension cables. A new CT scan was obtained and merged with the preoperative MRI to confirm that ventral contacts (1,5 or 2,6) were in the region of the LHA. Postoperative programming sessions were carried out until postoperative day 5, when patients underwent the second stage of the procedure. This step was conducted under general anesthesia. It involved the removal of external wires and the connection of leads to a pulse generator (IPG; Libra XP6644; St Jude Medical) via extension cables (model 6345; St Jude Medical).

Location of electrode contacts

To study the location of the contact used for chronic stimulation relative to internal anatomical landmarks, postoperative CT and preoperative T1 images were morphed to the USP- Würzburg atlas of the Human Brain [2]. This atlas was chosen because it co-registers histological sections and *post mortem in-situ* MRI of the same specimens. For histological processing, brains were fixed in formalin, embedded in celloidin, cut

serially and stained with gallocyanin. Co-registration of histological sections and imaging series was conducted with a previously described computational pipeline [2]. Images were normalized to MNI standard space (ICBM152) using the Advanced Normalization Tools (ANTs) Symmetric diffeomorphic (SyN) algorithm [3] and the transforms applied to the histological volume. Electrode trajectory and the volume of tissue activated (VTA) for each patient were calculated using the finite element method based VTA model described by Horn and colleagues [4] within Lead DBS [5]. Scaled electrode models were built in 3Dsmax 7 (Autodesk Inc., USA) and imported to Amira (v 5.4.1, Visage Imaging GmbH, Germany).

eReferences

1. Talakoub O, Paiva RR, Milosevic M, et al. Lateral hypothalamic activity indicates hunger and satiety states in humans. *Ann Clin Transl Neurol.* 2017;4(12):897-901.
2. Alho EJL, Alho A, Grinberg L, et al. High thickness histological sections as alternative to study the three-dimensional microscopic human sub-cortical neuroanatomy. *Brain Struct Funct.* 2017.
3. Avants BB, Epstein CL, Grossman M, Gee JC. Symmetric diffeomorphic image registration with cross-correlation: evaluating automated labeling of elderly and neurodegenerative brain. *Med Image Anal.* 2008;12(1):26-41.
4. Horn A, Reich M, Vorwerk J, et al. Connectivity Predicts deep brain stimulation outcome in Parkinson disease. *Ann Neurol.* 2017;82(1):67-78.
5. Horn A, Kuhn AA. Lead-DBS: a toolbox for deep brain stimulation electrode localizations and visualizations. *Neuroimage.* 2015;107:127-135.

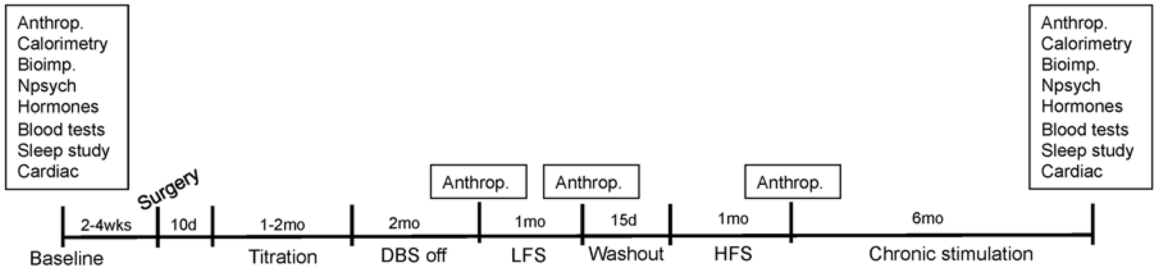

eFigure. Study timeframe. Baseline evaluations were collected 2-4 weeks prior to surgery. Ten days after the procedure, patients returned to the clinic and programming was started in a titration phase that lasted 1-2monts. DBS was then turned "off" for 2 months, either due to side effects or to standardize the study timeline across patients. After this phase, stimulation was delivered at low (LFS; 40Hz) or high frequencies (HFS; 130Hz) for 1 month, with 15 days washout in between. Thereafter, DBS was given to the patients for 6 months. Anthropometric measures (Anthrop) were collected at baseline and at the end of "off", LFS, HFS and chronic stimulation phases. At baseline and at the end of the chronic stimulation phase, patients underwent calorimetry, bioimpedantiometry (Bioimp), neuropsychological testing (Npsych), and sleep studies. Hormonal levels, cardiac tests and blood work-up were studied. During all follow-up evaluations and whenever necessary, patients underwent clinical and psychiatric assessments. Side effects were noted every time patients came to the clinic.

eTable 1. Pre- and postoperative hormonal levels.

	GH (ng/mL)	IGF1 (ng/mL)	Prolactin (ng/mL)	Free T4 (ng/dL)	TSH (mU/L)	LH (U/L)	FSH (U/L)	Testosterone* (ng/dL)	Estradiol* (pg/mL)	Cortisol (ug/dL)	ACTH (pg/mL)
**Baseline**	0.06 (0.05)	146.5 (47.0)	9.95 (2.4)	1.21 (0.1)	3.14 (0.7)	2.95 (2.7)	9.5 (8.6)	83.5 (75.7)	20.6 (1.9)	12.45 (4.4)	21.2 (8.2)
**6mo f-up**	0.05 (0.02)	158.5 (27.7)	25.6 (34.5)	1.32 (0.2)	2.66 (1.2)	4.05 (2.6)	15.4 (10.2)	49.5 (24.7)	27.0 (5.6)	7.40 (1.8)	19.4 (11.4)

GH- growth hormone; IGF-1- insulin-like growth factor 1; free T4- free thyroxin; TSH- thyroid-stimulating hormone; LH- luteinizing hormone; FSH- follicle stimulating hormone; ACTH- adrenocorticotropic hormone.

Values represent mean and standard deviation (in parenthesis). * testosterone and estradiol were only collected in males and females, respectively.

eTable 2. Pre- and postoperative levels of lipids, markers of hepatic function, glucose, insulin, vitamin D and uric acid.

	Cholesterol (mg/dL)	HDL (mg/dL)	LDL (mg/dL)	Triglycerides (mg/dL)	Glucose (mg/dL)	Insulin (mU/mL)	AST (U/L)	ALT (U/L)	GGT (U/L)	25 vit D (ng/dL)	Uric Acid (mg/dL)
**Baseline**	189.0 (11.2)	48.0 (7.6)	120.0 (13.7)	120.8 (20.1)	89.8 (9.0)	13.7 (7.9)	19.8 (7.4)	19.0 (6.5)	15.2 (6.4)	20.0 (1.8)	4.8 (1.1)
**6mo f-up**	172.0 (41.7)	49.0 (12.8)	116.3 (40.1)	93.0 (26.9)	88.3 (6.9)	13.4 (6.0)	20.3 (5.7)	31.3 (12.7)	18.3 (9.0)	23.7 (12.2)	5.0 (1.3)

HDL-high-density lipoproteins; LDL- low-density lipoproteins; AST- aspartate transaminase; ALT- alanine transaminase; GGT- gamma-glutamyl transferase; 25-vitD- 25-hydroxyvitamin D. Values represent mean and standard deviation (in parenthesis).

eTable 3. Pre- and postoperative sleep studies.

	Polysomnography (AHI)	Sleep efficiency (%)	REM latency (min)	Mean O_2 saturation (%)
**Baseline**	7.4 (3.4)	86.8 (7.2)	136.4 (46.4)	92.7 (1.0)
**6mo f-up**	7.2 (3.2)	85.8 (5.8)	152.5 (110.2)	92.0 (0.4)

AHI- apnea-hypopnea index (number of apnea/hypopnea events per hour); % sleep efficiency (% time spent asleep during the study); REM latency (time from sleep onset to the first epoch of rapid eye movement sleep). Values represent mean and standard deviation (in parenthesis).

eTable 4. Pre- and postoperative neuropsychological assessments.

Neuropsychological Test	Patient 1		Patient 2		Patient 3		Patient 4
	pre	post	pre	post	pre	post	pre*
WASI – total estimated IQ	58	57	67	74	64	73	45
WASI – verbal IQ	72	69	80	78	61	68	56
WASI – performance IQ	50	51	61	79	73	83	45
FAB – total score	10	8	15	12	13	11	2
Digit Span – forward	4	4	6	6	3	4	1
Digit Span – backward	3	2	5	5	3	4	0
Corsi Span– forward	2	4	4	6	3	5	2
Corsi Span– backward	3	2	4	2	4	5	0
GPT – DH (seconds)	>300	258	113	116	130	127	>300
GPT – NDH (seconds)	>300	285	144	114	146	136	>300
CVLT – total score	34	42	42	33	44	43	32

WASI-Wechsler Abbreviate Scale of Intelligence; IQ- Intelligence Quotient; FAB-Frontal Assessment Battery; Digit Span- Digit Span Memory Test; Corsi Span- Corsi Block Task; GPT- Grooved Pegboard Test; DH- Dominant Hand; DNH- Non-dominant hand; CVLT- California Verbal Learning Test. Postoperative assessments were conducted after 6months of chronic stimulation.

* Patient 4 had an estimated IQ of 45. Combined with poor language skills this precluded the conduction of other neuropsychological tests. Based on this and other preoperative results, no post-surgical assessments were conducted.

eTable 5. Individualized pre and postoperative results of anthropometry, bioimpedanciometry and calorimetry

**Patient 1**	Baseline	DBS off	LFS	HSF	6mo f-up
Weight (Kg)	136.0	136.5 (0.4%)	137.0 (0.7%)	136.5 (0.4%)	140.0 (2.9%)
Body Mass Index (Kg/m^2)	56.0	50.7 (-9.5%)	50.9 (-9.1%)	50.7 (-9.5%)	52 (-7.1%)
Abdominal circumf. (cm)	145	144 (-0.7%)	142 (-2.1%)	144 (-0.7%)	146 (0.7%)
Neck circumf. (cm)	44	44 (0%)	42 (-4.5%)	44 (0%)	43 (-2.3%)
Bioimpedanciometry (% fat)	54.4				56 (2.9%)
Calorimetry (kcal)	2074				2212 (6.7%)
**Patient 2**	Baseline	DBS off	LFS	HSF	6mo f-up
Weight (Kg)	85.0	96.2 (13.2%)	90.7 (6.7%)	99.2 (16.7%)	109.0 (28.2%)
Body Mass Index (Kg/m^2)	33.6	38.0 (13.1%)	35.8 (6.5%)	39.2 (16.7%)	43.1 (28.3%)
Abdominal circumf. (cm)	108	125 (15.7%)	116 (7.4%)	123 (13.9%)	129 (19.4%)
Neck circumf. (cm)	32	33 (3.1%)	33.5 (4.7%)	35 (9.4%)	37 (15.6%)
Bioimpedanciometry (% fat)	50				54 (8.0%)
Calorimetry (kcal)	1827				1725 (-5.6%)
**Patient 3**	Baseline	DBS off	LFS	HSF	6mo f-up
Weight (Kg)	82.0	83.4 (1.7%)	84.8 (3.4%)	85 (3.7%)	85.0 (3.7%)
Body Mass Index (Kg/m^2)	32.2	32.7 (1.6%)	33.3 (3.4%)	33.4 (3.7%)	33.4 (3.7%)
Abdominal circumf. (cm)	111	113 (1.8%)	115 (3.6%)	116 (4.5%)	114 (2.7%)
Neck circumf. (cm)	38	37.5 (-1.3%)	38 (0%)	38.5 (1.3%)	38 (0%)
Bioimpedanciometry (% fat)	49				51 (4.1%)
Calorimetry (kcal)	1636				1580 (-3.4%)
**Patient 4**	Baseline	DBS off	LFS	HSF	6mo f-up
Weight (Kg)	76.8	79.4 (3.4%)	77.8 (1.3%)	80.8 (5.2%)	82.2 (7.0%)
Body Mass Index (Kg/m^2)	36.5	32.7(-10.4%)	37 (1.4%)	38.4 (5.2%)	39.0 (6.8%)
Abdominal circumf. (cm)	100	112 (12%)	110 (10%)	111 (11.0%)	114 (14.0%)
Neck circumf. (cm)	30	31 (3.3%)	37 (23.3%)	37.5 (25.0%)	32 (6.7%)
Bioimpedanciometry (% fat)	46				49 (6.5%)
Calorimetry (kcal)	1519				1542 (1.5%)

Circumf- circumference; F-up- follow up; LFS- low frequency stimulation; HFS- high frequency stimulation. Values represent mean and percent change from baseline (in parenthesis).

eTable 6. Individualized measures of pre- and postoperative hormonal levels.

		GH (ng/mL)	IGF1 (ng/mL)	Prolactin (ng/mL)	Free T4 (ng/dL)	TSH (mU/L)	LH (U/L)	FSH (U/L)	Testosterone (ng/dL)	Estradiol (pg/mL)	Cortisol (ug/dL)	ACTH (pg/mL)
**Pt 1**	**Bsl**	0.03	213	9.9	1.17	2.35	0.2	1.6	30		12.3	26
	**6mo**	0.03	198	7.5	1.26	2.05	6.1	23.3	32		6.2	20.7
**Pt 2**	**Bsl**	0.12	146	7	1.37	3.9	1.2	3.9		19.2	18.6	9
	**6mo**	0.04	135	5.6	1.58	1.86	1.4	5.2		30.9	5.8	5.2
**Pt 3**	**Bsl**	0.07	117	12.8	1.1	3.54	6	20.6	137		10.6	24.3
	**6mo**	0.06	156	12	1.13	2.33	6.4	25	67		8	18.81
**Pt 4**	**Bsl**	0.02	110	10.1	1.18	2.76	4.4	11.9		21.9	8.3	25.5
	**6mo**	0.06	145	77.3	1.3	4.4	2.3	8		23	9.6	33

Pt- patient; Bsl- Baseline; 6mo- 6months follow up; GH- growth hormone; IGF-1- insulin-like growth factor 1; free T4- free thyroxin; TSH- thyroid-stimulating hormone; LH- luteinizing hormone; FSH- follicle stimulating hormone; ACTH- adrenocorticotropic hormone.

eTable 7. Individualized measures of pre- and postoperative levels of lipids, markers of hepatic function, glucose, insulin, vitamin D and uric acid.

		Cholesterol (mg/dL)	HDL (mg/dL)	LDL (mg/dL)	Triglycerides (mg/dL)	Glucose (mg/dL)	Insulin (mU/mL)	AST (U/L)	ALT (U/L)	GGT (U/L)	25 vit D (ng/dL)	Uric Acid (mg/dL)
**Pt 1**	**Bsl**	191	53	116	110	87	16.4	16	19	12	22	5.9
	**6mo**	192	68	106	89	97	13.4	20	44	13	21	5.7
**Pt 2**	**Bsl**	181	49	106	130	86	6.5	30	28	9	18	3.3
	**6mo**	133	43	104	72	85	6.1	13	15	11	12	3.2
**Pt 3**	**Bsl**	204	37	138	144	103	23.7	20	16	23.7	21	4.9
	**6mo**	221	40	174	132	90	20.8	27	38	31	41	6
**Pt 4**	**Bsl**	180	53	120	99	83	8.2	13	13	16	19	5
	**6mo**	142	45	81	79	81	13.4	21	28	20	20.8	4.9

Pt- patient; Bsl- Baseline; 6mo- 6months follow up; HDL-high-density lipoproteins; LDL- low-density lipoproteins; AST- aspartate transaminase; ALT- alanine transaminase; GGT- gamma-glutamyl transferase; 25-vitD- 25-hydroxyvitamin D.
